# Harnessing antibody-mediated recognition of the intracellular proteome with T cell receptor-like specificity

**DOI:** 10.3389/fimmu.2024.1486721

**Published:** 2024-11-22

**Authors:** Maya Haus-Cohen, Yoram Reiter

**Affiliations:** Laboratory of Molecular Immunology and Immunotherapy, Faculty of Biology Technion – Israel Institute of Technology, Haifa, Israel

**Keywords:** immunotherapy, CAR- T cells, TCR - T cell receptor, TCR-like antibodies (TCRL), bi-specific antibodies

## Abstract

The clinical success of cancer immunotherapy has driven ongoing efforts to identify novel targets that can effectively guide potent effector functions to eliminate malignant cells. Traditionally, immunotherapies have focused on surface antigens; however, these represent only a small fraction of the cancer proteome, limiting their therapeutic potential. In contrast, the majority of proteins within the human proteome are intracellular, yet they are represented on the cell surface as short peptides presented by MHC class I molecules. These peptide-MHC complexes offer a vast and largely untapped resource for cancer immunotherapy targets. The intracellular proteome, including neo-antigens, presents an exciting opportunity for the development of novel cell-based and soluble immunotherapies. Targeting these intracellular-derived peptide-MHC molecules on malignant cell surfaces can be achieved using specific T-cell receptors (TCRs) or TCR-mimicking antibodies, known as TCR-like (TCRL) antibodies. Current therapeutic strategies under investigation include adoptive cell transfer of TCR-engineered or TCRL-T cells and CAR-T cells that target peptide-MHC complexes, as well as soluble TCR- and TCRL-based agents like bispecific T cell engagers. Recent clinical developments in targeting the intracellular proteome using TCRL- and TCR-based molecules have shown promising results, with two therapies recently receiving FDA approval for the treatment of unresectable or metastatic uveal melanoma and synovial sarcoma. This review focuses on the processes for selecting and isolating TCR- and TCRL-based targeting moieties, with an emphasis on pre-clinical and clinical studies that explore the potential of peptide-MHC targeting agents in cancer immunotherapy.

## 1 Introduction

When it comes to therapeutic interventions, the cell surface and intracellular proteomes represent distinct but equally vital landscapes for drug discovery and development. Understanding the differences between these proteomes is essential for designing effective treatments, particularly in the context of diseases such as cancer, autoimmune disorders, and infectious diseases.

The cell surface proteome, comprising proteins that reside on the outer membrane of cells, is the most accessible part of the cell’s molecular machinery. These proteins are directly exposed to the extracellular environment, making them prime candidates for therapeutic targeting. Receptors, transporters, and adhesion molecules on the cell surface play critical roles in cell communication, nutrient uptake, and immune recognition. Therapies targeting the cell surface proteome have seen considerable success, particularly in cancer treatment. Monoclonal antibodies, such as those targeting HER2 in breast cancer, bind to specific surface proteins, blocking signals that promote tumor growth or flagging cells for destruction by the immune system. Similarly, CAR-T cell therapies harness the immune system by engineering T cells to recognize and attack cancer cells expressing specific surface antigens. The ease of accessibility and the critical functions these proteins perform make the cell surface proteome a rich source of therapeutic targets.

In contrast, the intracellular proteome consists of proteins within the cell, including those in the cytoplasm, nucleus, and various organelles. These proteins are involved in a myriad of essential processes, such as metabolism, gene expression, and intracellular signaling. Although these proteins are not directly accessible from outside the cell, they are equally crucial in disease pathology.

Targeting the intracellular proteome poses significant challenges due to the need to penetrate the cell membrane to reach these proteins. Comprehensive cancer proteomics studies reveal that in many of the major cancer types over 500 genes involved in the transformation of normal cells into cancerous ones, with nearly half of these genes coding for intracellular proteins. Targeting intracellular proteins with these therapies remains challenging and there is a critical need to develop novel strategies for targeting intracellular proteins effectively.

A promising strategy to target the intracellular proteome involves developing antibodies that target peptides derived from intracellular antigens. Intracellular proteins are degraded by the proteasome into short peptides, typically 8–10 amino acids long, which are presented on the cell surface by major histocompatibility complex class I (MHC-I) molecules, also known as the human leukocyte antigen (HLA) system in humans (1, 2). Some peptide/MHC complexes (pMHC) are implicated in various cancers and can be targeted by T cell receptor (TCR) therapy. These complexes also serve as potential targets for antibody development. Antibodies that recognize pMHC complexes, known as TCR mimic or TCR-like antibodies, mimic the ability of TCRs on T cells to recognize these complexes. The peptides presented on these complexes may originate from a variety of intracellular tumor antigens, including viral oncogene products, transcription factors, oncofetal proteins, cancer-testis antigens, or neoantigens derived from mutated oncogenes. TCRm antibodies significantly expand the range of therapeutic targets and hold considerable clinical promise. This review will focus on the development of TCR-like antibodies targeting pMHC complexes for cancer immunotherapy.

## 2 The origin of cellular internal targets

Intracellular-derived targets for immunotherapy, such as those targeted by CAR-T cells, typically originate from proteins that are processed and presented on the surface of cells via major histocompatibility complex (MHC) molecules. These proteins can be sourced from various cellular processes, including the normal turnover of proteins, misfolded proteins, or those associated with disease states like cancer. In the context of cancer, for instance, tumor cells often present abnormal or overexpressed proteins that can be recognized as antigens. These tumor-associated antigens (TAAs) or neoantigens, which arise from mutations within the tumor’s genetic material, can be processed intracellularly and displayed on the cell surface as peptide-MHC complexes. This presentation enables the immune system, particularly T cells engineered to recognize these specific complexes, to distinguish between healthy cells and those that are cancerous or infected, leading to targeted immune responses. Adaptive immune surveillance mechanisms relay on cell surface presentation of MHC class I and II molecules, bound to peptides derived from either intracellular or extracellular proteins, respectively. Presentation of non-self-intracellular peptides, on MHC class I molecules to CD8+ cytotoxic T cells via a T cell receptor (TCR), allows the recognition of viral infected or malignant cells, resulting in target-specific immune response, aiming for abnormal cell elimination (1).

All nucleated cells express MHC class I molecules, a heterodimer containing a non-polymorphic β2 macroglobulin (β2M) light chain and a polymorphic heavy chain, encoded in humans by three duplicated genes on chromosome 6, named HLA-A, HLA-B, and HLA-C (2). The MHC class I heavy chain was found to be the most polymorphic gene in humans, where each individual expresses a unique set of six HLA heavy chains out of over 20,000 polymorphic alleles (3).

Assembled MHC class I heterodimers mainly differ from one another in their peptide binding groove domains, resulting in a variable set of peptides that, based on their charge and length, may fit a certain MHC class I variant, but not others (4). Accordingly, certain diseases were found to be positively correlated with specific MHC class I alleles, such as HLA-B*27:05 in ankylosing spondylitis (5). Moreover, a change in a single amino acid within the peptide binding groove may completely alter the HLA presented peptide repertoire. For example, a dramatic change in peptide binding repertoire was observed between HLA-B*15:01 and HLA-B*15:18 alleles, which only differ in a single amino acid (6). Of note, some HLA class I heavy chains were found to be more abundant than others, such as the presentation of HLA-A2 allele in approximately 50% of Caucasian humans (7).

Proteins that originated in either the cytosol or nucleus are degraded by the 26S proteasome, followed by presentation on MHC class I molecules (8). The source of 26S degraded peptides includes (a) defective ribosomal products (DRIPs), consisting mainly of misfolded proteins and prematurely terminated polypeptides degraded in close to synthesis time; (b) proteins at the end of their lifetime; and (c) signal sequence fragments. 26S Degraded peptides translocate to the ER lumen by transporter associated with antigen processing (TAP), where they interact with the MHC class I molecule (9).

Assembly of peptide/MHC class I complex is a multistep process, starting with the temporally stabilization of the MHC class I heterodimer, containing the β2M light chain and the variable heavy chain, by ER chaperones such as calreticulin, ERp57, PDI, and tapasin, creating the peptide loading complex (PLC) (10). Then, the usually 8–12 amino acid (aa) long peptide bound to TAP interacts with tapasin, delivering the peptide to the ER lumen. Release of all MHC chaperones occurs if the delivered peptide binds the MHC class I peptide binding groove with sufficient affinity, creating a stable peptide/MHC class I complex (11). During assembly, a certain MHC class I molecule may switch several different peptides, resulting in a conformational change in each peptide exchange. ER release and transport to the cell surface occur when a high affinity peptide binds the MHC binding groove, creating a stable peptide/MHC class I complex (12). Peptides that failed to stabilize the MHC class I molecule are transformed back to the cytosol for full degradation via the ER-associated degradation (ERAD) pathway (13).

Presentation of peptide/MHC class I molecules on the cell surface was found to be influenced by several factors, such as protein copy number, intracellular conditions, HLA heavy chain allele variants, and cell type (1, 13). Proteins expressed at a copy number lower than 1000 may not be presented on MHC class I molecules owing to their relatively low expression. Consequently, these proteins, especially if unstable, may evade MHC-based immune surveillance system (1). Moreover, different intracellular conditions may affect protein expression, such as upregulation of protein processing and MHC class I expression in the presence of IFNγ and enhanced protein degradation in irradiated treated cells, affecting peptide repertoire presented on the cell surface (14). Of note, the cell surface presentation level of MHC class I molecules varies between 10,000 and 500,000 molecules, depending on cell type. For example, professional antigen presenting cells, such as DCs, express high MHC class I levels, while some malignant cells downregulate MHC class I presentation, escaping immune surveillance (15, 16).

## 3 Directing therapeutic action toward intracellular targets

In the realm of modern medicine, targeting intracellular processes has emerged as a highly promising strategy for developing effective therapies. Unlike traditional approaches that focus on extracellular targets such as cell surface receptors or circulating proteins, directing therapeutic action toward intracellular targets addresses the root causes of many diseases at the cellular level. This paradigm shift allows for more precise intervention, potentially leading to more effective and specific treatments with fewer off-target effects.

The rationale for intracellular targeting lies in the fact that intracellular targets are often implicated in various disease mechanisms, including cancer, neurodegenerative disorders, and genetic diseases. These targets can include proteins involved in signaling pathways, enzymes responsible for metabolic processes, or mutated genes contributing to disease pathology. By focusing on intracellular targets, therapeutic strategies aim to modulate or correct these processes directly, offering the potential to achieve more substantial and sustained therapeutic outcomes. The majority of intracellular targets, are proteins or enzymes that play crucial roles in cellular functions, for example, mutated or overexpressed proteins in cancer cells can be targeted by small molecules or monoclonal antibodies to inhibit their activity.

Targeting intracellular processes presents several challenges, mainly the one related to specificity. Ensuring that therapies selectively target disease-associated molecules without affecting normal cellular functions is crucial to minimize side effects and improve efficacy. In addition, similar to other therapeutic strategies, the development of resistance to intracellular targeting approaches is a concern. Understanding the mechanisms of resistance and developing strategies to overcome them is essential for long-term success.

Interaction between T cell receptor (TCR) and cell surface foreign or abnormal peptides, in the context of MHC class I molecules, provides one of the first and most critical steps in adaptive immune response (17). Mature αβ T cells express a disulfide linked heterodimeric TCRs, consisting of the TCRα and TCRβ chains (18). Unique TCR sequences are assembled owing to somatic recombination in variable (V), diversity (D), and joining (J) regions, creating the CDR1, CDR2, and CDR3 domains of the TCR antigen binding site (19). Recognition and elimination of specific malignant and infected cells by the adaptive immune arm, through the unique TCR-pMHC interaction, is dependent on a variety of molecular and cellular features such as variation in MHC alleles, intracellular processing by the proteasome, peptide presentation, and TCR clonality (18). TCR and pMHC complex interactions induce intracellular T cell signaling via immune-receptor tyrosine-based activation motif (ITAMs), located on the CD3 chains expressed as part of the TCR complex. Affinity between TCR and pMHC complex affects the intracellular signaling level, thereby influencing T cell faith. For example, it was found that a strong interaction of CD8+ TCRs with pMHC molecules usually results in robust T cell proliferation, while weak interaction results in CD8+ memory T cell formation (20).

Targeting an intracellular abnormal protein-derived peptide, expressed in the context of MHC class I molecule, can be achieved using engineered T cells, manipulated to express specific TCRα and TCRβ genes, with specificity toward a desired pMHC complex. In this case, target cells must express the peptide of interest in the context of a specific MHC class I variant, as engineered TCR recognizes both peptide and MHC class I molecules. Accordingly, TCR-based treatments focus on relatively abundant MHC class I alleles, such as HLA-A*02:01 (21). Tumor targeting TCR sequences can be identified via isolation and deep sequencing strategies of tumor infiltrating lymphocytes (TILs). These TIL CD8+ lymphocytes, present in the tumor environment, are sorted as single cells, followed by sequencing and TCRα-TCRβ pairing analysis. Alternatively, tetrameric cancer-related peptide-MHC molecules can be used to identify peripheral blood lymphocytes (PBLs) expressing TCRs in diseased patients. These TCRs can further be cloned and re-expressed in cytotoxic T lymphocytes (CTLs) derived from peripheral blood, creating tumor-specific T cells (22, 23). For example, TCRs targeting melanoma-associated antigen recognized by T cells (MART-1) in the context of HLA-A2 derived from melanoma metastatic patient TILs were cloned and transduced into activated T cells, showing anti-tumor activity against HLA-A2+ melanoma cells (24). Optimization of TCR affinity toward a desired antigen also improved the therapeutic potential of TCR-based treatment, such as the affinity optimized AFP/HLA-A2 targeting TCR, showing improved activity against liver malignant cells (25).

**Figure 1:**
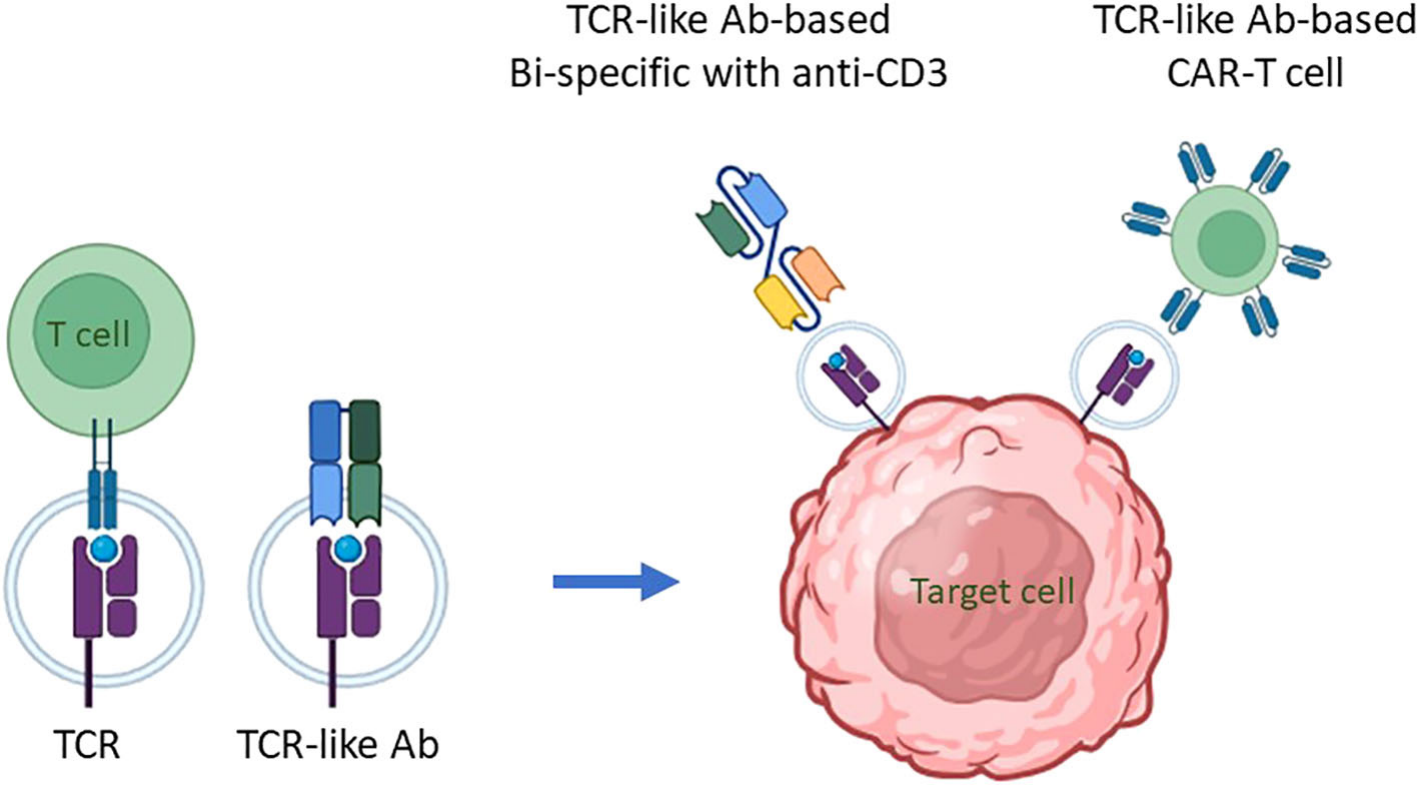
The principle of TCR-like antibodies and their therapeutic mode of action. Intracellular proteins undergo proteasomal degradation, breaking down into smaller peptide fragments, usually 8-10 amino acids in length. These peptide fragments are then transported into the endoplasmic reticulum, where they are loaded onto major histocompatibility complex class I (MHC I) molecules, known as human leukocyte antigens (HLA) in humans, for presentation on the cell surface. This process is critical for immune surveillance, as it allows T cells to monitor cellular health by recognizing peptide/MHC (pMHC) complexes displayed on cell surfaces. The T-cell receptor (TCR) on T cells specifically binds to these pMHC complexes, enabling the immune system to detect and respond to abnormal cells, including those undergoing transformation into cancerous states. This recognition process can be mimicked for therapeutic purposes through the development of antibodies that specifically target pMHC complexes. These are termed TCR mimic, or TCR-like, antibodies because they can recognize pMHC complexes similarly to TCRs, making them potent agents for targeting intracellular antigen-derived peptides presented by MHC I molecules on malignant cells. TCR-like antibodies can be employed in cell-based immunotherapy to target malignant cells presenting specific pMHC complexes. Two primary immunotherapeutic strategies have been developed using these antibodies: (i) T Cell Engagers: In this approach, a TCR-like antibody is engineered to include an anti-CD3 effector moiety, creating a bispecific T-cell engager (BiTE). The BiTE molecule binds to both the target pMHC complex on malignant cells and CD3 on T cells, effectively redirecting T cells toward cancer cells and stimulating a cytotoxic response against the targeted tumor cells. (ii) CAR-T Cells Expressing TCR-Like Antibodies: In this strategy, T cells are genetically engineered to express a chimeric antigen receptor (CAR) that incorporates a TCR-like antibody component. This CAR is specifically designed to recognize the pMHC complex associated with the target antigen. When these CAR-T cells encounter a malignant cell displaying the appropriate pMHC complex, they are activated to initiate an immune response, resulting in the targeted killing of the cancer cell. By employing TCR mimic antibodies in these ways, immunotherapies can effectively target intracellular tumor antigens that would otherwise be inaccessible, expanding the scope of antigens available for cancer immunotherapy. These strategies hold particular promise for treating malignancies where conventional therapies or surface-targeting antibodies have shown limited efficacy, thereby advancing the potential for precise and potent elimination of cancer cells.

Mimicking adaptive immune response surveillance system to target abnormal peptide presentation can also be achieved using T-cell-receptor-like (TCRL) antibodies, also termed T cell receptor mimic (TCRm) antibodies. In this approach, combining the advantages of the both humoral and cellular adaptive immune response, antibodies mimicking the binding of a TCR to pMHC class I complex are used to target abnormal peptides presented on the cell surface of malignant or infected cells with nanomolar affinities (26, 27). The antigen binding domain of these antibodies can further be cloned to create fusion molecules, such as immunocytokines, bispecific T-cell engager (BiTE), and chimeric antigen receptor (CAR)-T constructs (28); see **Figure 1** schematics for the principle of TCR-like antibodies and their therapeutic mode of action.

**Figure 2:**
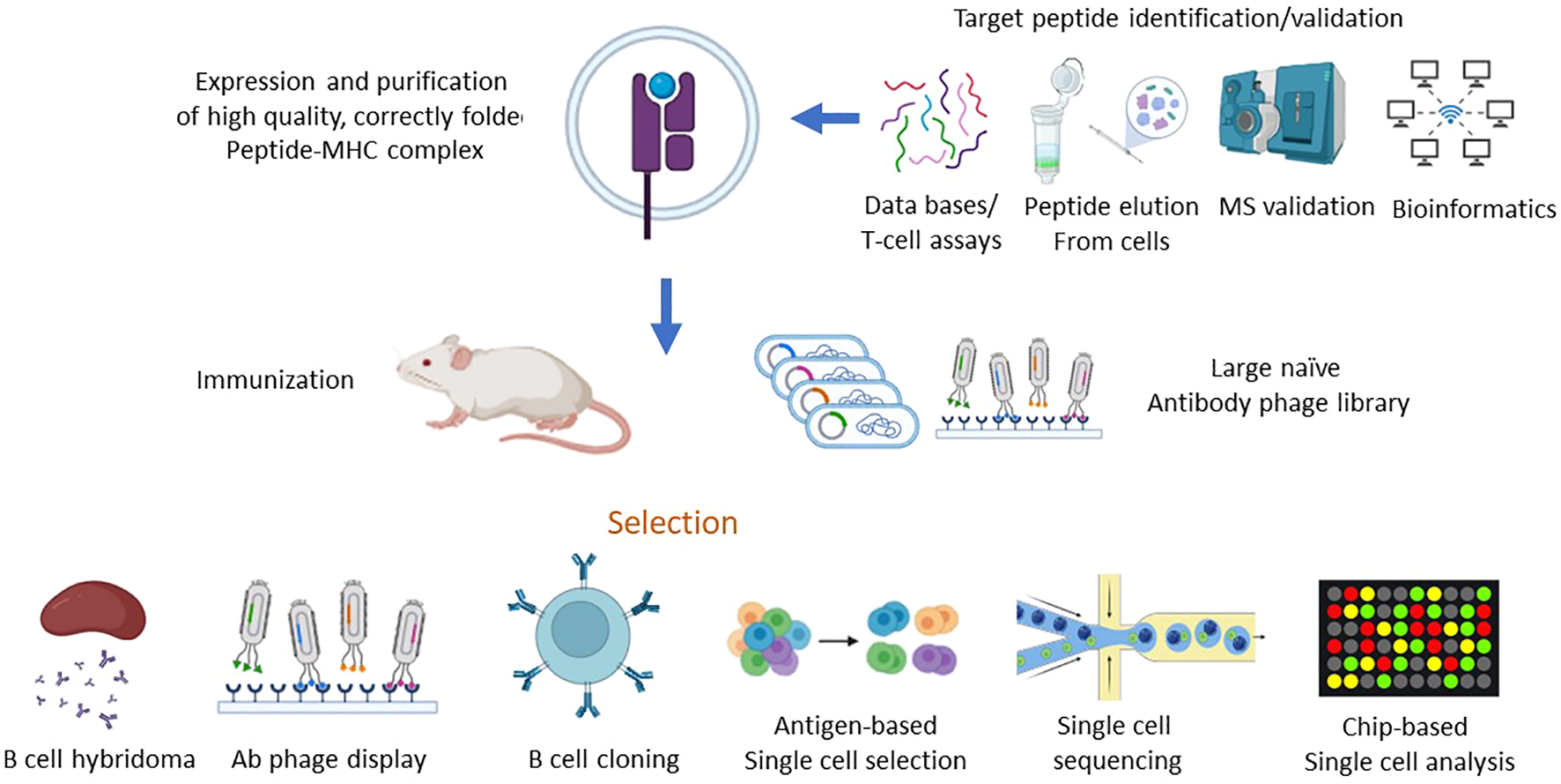
Schematic of strategies for isolation of TCR-like antibodies. The process of isolating TCR-like (TCRL) antibodies involves a multi-step approach that starts with the identification of target peptides bound to MHC molecules, followed by the generation of purified peptide-MHC (pMHC) complexes. These complexes serve as the key immunogens in various antibody isolation platforms, including phage display and *in vivo* immunization, ultimately leading to the production of TCR-like antibodies capable of recognizing specific peptide-MHC complexes with high affinity and specificity. 1.Target Peptide Identification and Validation: To pinpoint suitable target peptides, various strategies are employed to identify and validate peptides bound to MHC molecules, which are indicative of intracellular proteins presented on the cell surface. Key methods include: HLA Peptidomics Databases: These databases provide extensive data on peptides naturally presented by different HLA alleles, offering a foundation for identifying relevant tumor-associated or disease-specific antigens. Peptide Elution and Mass Spectrometry: This approach involves eluting peptides directly from the surface of target cells, followed by analysis via mass spectrometry (MS) to accurately determine peptide sequences and assess potential immunogenicity. Peptide Prediction Algorithms: Computational algorithms predict the binding affinity of peptides to specific HLA alleles, streamlining the process of selecting candidates with high likelihoods of immunogenicity. HLA Peptidome Bioinformatics Tools: These tools assist in analyzing HLA peptide repertoires, providing insights into peptide processing and presentation specific to disease states or cell types. T Cell Assays: Functional assays using T cells can confirm the immunogenic potential of peptides, further validating their relevance as potential targets for TCRL antibodies. 2.Generation of Peptide-MHC Complexes: Once a target peptide has been identified, it is synthesized and used to produce high-quality, correctly folded peptide-MHC complexes. The generation process involves: Recombinant Expression of MHC Components: The MHC heavy chain and β2-microglobulin (β2m) are produced in bacterial expression systems, either as single-chain or double-chain formats. *In Vitro* Refolding: Following recombinant expression, the MHC heavy chain and β2m are refolded *in vitro* along with the target peptide. This step is critical to ensure that the peptide-MHC complex is presented in a stable and biologically relevant conformation suitable for subsequent TCRL antibody selection. 3. Isolation of TCR-like Antibodies: With the target pMHC complex in hand, TCR-like antibodies are isolated through several methodologies, such as: Phage Display Libraries: Libraries of antibody-displaying phages are screened against the pMHC complex, allowing for high-throughput selection of clones with specific affinity to the target complex. Naïve and Targeted Phage Libraries: Either large, naïve libraries or libraries constructed from immunized animals are employed, depending on the availability of pre-existing immune responses. *In Vivo* Immunization: Recombinant pMHC molecules or pMHC-expressing cells can be used to immunize animals, generating an immune response specifically directed against the pMHC target. Direct B Cell Selection and Cloning: In this method, antigen-specific B cells are directly isolated using flow cytometry or chip-based single-cell analysis, followed by deep sequencing to identify and clone antibodies with specificity for the pMHC complex. 4. Selection and Screening of TCR-like Antibodies: After initial selection, further refinement is required to isolate TCRL antibodies with the desired specificity, affinity, and functionality: Primary and Secondary Screening of Hybridoma Clones: Hybridoma cells producing antibodies can be screened in multiple rounds to ensure optimal specificity and binding strength to the target pMHC. Flow Cytometry and Single-Cell Analysis: Techniques such as flow cytometry sorting or chip-based analysis enable precise isolation of single cells that recognize the target pMHC complex. Deep sequencing provides a high-throughput means of identifying unique antibody sequences from these cells. The systematic approach to isolating TCR-like antibodies combines robust peptide identification, efficient generation of pMHC complexes, and diverse selection strategies. The resulting TCRL antibodies have the potential to recognize intracellular antigens presented by MHC molecules, offering promising avenues for targeted immunotherapy applications, particularly in cancer and infectious diseases.

The first step in TCRL isolation usually requires the expression and purification of the target pMHC of interest. This can be achieved by either expression of single chain trimers (SCTs), which is a single polypeptide consisting of all three subunits of the pMHC class I molecule including β2M, HLA heavy chain, and the peptide of interest, all connected with flexible linkers, or by β2M and HLA heavy chain linked covalently with a flexible linker, expressed in E. coli, and subsequently refolded with the desired MHC-restricted peptide. Alternatively, MHC class I–peptide complexes can also be generated using separate β2M and HLA heavy chains, expressed in E. coli, followed by refolding with a synthetic peptide, thus obtaining the full tetrameric pMHC class I–peptide complex. Once correctly folded, these constructs resemble the cognate pMHC class I molecules presented on cells, in a soluble form (29, 30). Traditional methods to isolate TCRL antibodies include scanning of phage display libraries or *in vivo* immunizations with the pMHC complex, followed by hybridoma or human B cell cloning assays. Using the phage display method, ScFV- or Fab-based libraries are scanned against monomeric soluble target pMHC molecules and control pMHC complexes. ScFV/Fab that showed specificity toward target pMHC can be further cloned into Full IgG abs (31). For example, the isolation of a TCRL ab against the tumor antigen WT1 in the context of HLA-A2 was achieved using ScFV-based phage display library scan (32). In TCRL isolation-based hybridoma assay, animals are immunized with specific pMHC molecule or antigen presenting cells (APCs), expressing the pMHC of interest (33). Vaccine triggered immune response may result in antibodies that are specific toward the pMHC molecule. Using vaccines based on pMHC expressing cells was found to be superior to recombinant soluble pMHC molecule vaccination, such as the isolation of TCRL against PRI/HLA-A2 antigen, tested in both soluble and cell vaccine-based approaches using Balb/c mice. Immunization with pMHC soluble molecules resulted in several pMHC targeting potential clones, while using vaccination-based PRI pulsed cells resulted in no potential clones (34). B cell cloning is another vaccine-based approach for the purpose of pMHC TCRL isolation. In this case, animals are immunized with the target of interest, followed by single-cell sorting of PBMCs, based on fluorophore labelled target tetramers and B cell markers. Ig primers are then used to amplify cDNAs derived from sorted cells, followed by cloning into full IgG sequences (35). Recently, Tatsuhiko et al. described a new chip-based TCRL isolation method. Here, pMHC rabbit immunization followed by single-cell chip-based selection of pMHC specific antibody producing cells derived from target organs was subjected to amplification of heavy and light cDNA, resulting in the isolation of specific TCRL abs (36, 37). The experimental strategies employed for the isolation of TCR-like antibodies are summarized in **Figure 2**.

## 4 Selecting the target pMHC

Selecting the target pMHC (peptide-MHC complex) is a critical step in designing effective immunotherapies, particularly in fields like CAR-T cell therapy and T-cell receptor (TCR) therapy. This process involves identifying and validating specific peptide-MHC complexes that can be recognized by engineered T cells to target and eliminate cancer cells or pathogens. Major Histocompatibility Complex (MHC) molecules present peptide fragments on the surface of cells. There are two main classes: Class I MHC molecules (which present peptides to CD8+ cytotoxic T cells) and Class II MHC molecules (which present peptides to CD4+ helper T cells). The peptides are short chains of amino acids derived from proteins that are processed and presented by MHC molecules. The specificity of T-cell recognition depends on the peptide-MHC (pMHC) complex. The types of peptide targets for clinical applications include: Tumor-Specific Antigens (TSAs): These are antigens that are expressed only on tumor cells and not on normal cells. Targeting TSAs can minimize off-target effects. For example, the targeted pMHC complex should preferably be a neo-antigen, expressed exclusively on malignant cells. These TSAs result from specific mutations in the malignant cells, thus targeting these unique and specific pMHC targets increases treatment safety by increasing on-target and reducing off-tumor responses. Tumor-Associated Antigens (TAAs) which are antigens that are overexpressed in tumor cells but also present in normal tissues, albeit at lower levels.

The challenge here is to minimize collateral damage to normal tissues. In the epitope selection process, one should consider: the binding affinity of the peptide, the peptide must bind with high affinity to the MHC molecule to ensure stable presentation on the cell surface; Immunogenicity, i.e., the peptide should be capable of eliciting a strong immune response. This includes considering the peptide’s ability to activate T cells effectively; and specificity: select peptides that are specific to the target cells to avoid cross-reactivity with non-target cells. Selecting the appropriate pMHC complex is a multifaceted process that requires careful consideration of the antigen’s specificity, immunogenicity, and the potential for off-target effects. Advances in immunology, molecular biology, and computational techniques continue to enhance our ability to identify and validate optimal targets, thereby improving the efficacy and safety of T-cell-based therapies.

Thus, the choice of p/MHC target for a TCRL- or TCR-based therapy is a key factor in the design and application of the therapeutic agent as it will dictate the delicate balance between an unmet need indication, efficacy, and the key issue of selectivity and specificity. For example, specific *in vitro* activity was detected in TCRs isolated from CML patients, directed against the crucial BCR-ABL mutation, resulting from a translocation between chromosome 22 and 9, found in approximately 95% of CML patients (38). Alternatively, tumor-associated antigens (TAAs), such as growth factors and cell cycle oncogenic proteins, which are known to be largely expressed on malignant cells, but their expression may be found in other tissues, can be targeted as well, while considering possible on-target but off-tissue response (39). Prediction of peptides formed by degradation of a protein of interest and their binding to MHC class I molecule can be predicted by dedicated algorithms (40). Alternatively, such TAAs and TSAs targets can be identified and characterized biochemically by elution of peptide presented on malignant cells in the context of MHC class I molecules, followed by mass spectrometry (33). The quest for new targets continues, with a current phase 1 clinical trial for the identification of somatic mutations and HLA typing in several solid tumors. Targeting a pMHC derived from a protein that has a key role in cell proliferation, such as growth factors, would potentially improve the chances that the target pMHC will not be lost or down modulated under selection pressure (33). Moreover, choosing a relatively abundant MHC class I allele is also a key factor in target selection, as treatment is relevant only in the context of the specific peptide on the specific MHC class I allele. Accordingly, as HLA-A2:01 was found to be the most abundant out of the HLA-A alleles in Caucasians, it is usually selected as the MHC allele in targeted TCRL- and TCR-based therapy (41). An improvement in TCR- or TCRL-CAR-based therapy can also be achieved by targeting high-density pMHC tumor-expressing cells, as the relative presentation of pMHC of interest on malignant cells was found to be positively correlated with T cell response (42).

TCR- and TCRL engineered T cells streamline practical challenges associated with TIL and T cell clone therapies, enabling efficient generation of potent cell products. Key advantages include: Simple autologous T cell procurement, e.g., via leukapheresis. Rapid production with pre-selected TCRs that optimize efficacy and specificity. Introduction of TCRs into minimally differentiated T cells with improved engraftment and proliferative capacity. Potential for concurrent genetic modifications to enhance T cell survival, resistance to inhibitory signals, and TCR signaling.

TCR clinical trials have targeted diverse classes of antigens and, in many cases, distinct epitopes derived from the same antigen. Clinical Trials include TCR therapies which target various antigens (see examples of targets and trials in **Table 1** and **Table 2**, respectively):

**Table 1:** Examples for targets used in preclinical studies of TCR and TCRL immunotherapies.

Target class	Examples	Tumor type
Overexpressed proteins	WT1	Leukemia, ovarian cancer, colon cancer, mesothelioma
	AFP	Hepatocellular carcinoma
	PRAME	Leukemia, lymphoma, melanoma, breast cancer, colon cancer
	MAGE	Melanoma
	NY-ESO-1	Melanoma
Fusion proteins	BCR-ABL	Chronic myeloid leukemia
	PML-RARa	Acute promyelocytic leukemia
Oncogenic viral antigens	EBV proteins	Burkitt's lymphoma, Hodgkin's lymphoma, and nasopharyngeal carcinoma
	HPV 16 E6/E7	Cervical, oral and oropharyngeal cancers
	CMV proteins	Breast, colon, prostate and glioblastoma
	HBV proteins	Hepatocellular carcinoma
Mutated antigens	Ras	Pancreatic, lung, and colorectal cancers
	p53	Breast, bladder, lung and brain tumors
	Myc	Breast, colorectal, pancreatic and gastric cancers

**Table 2:** Examples of targets in bispecific or CAR-T TCR and TCR-mimic clinical trials.

Antigen	HLA	TCR Vs. TCR-mimic	Disease
Tissue differentiation			
gp100	A*02:01	TCR	Melanoma, Uveal MEL
Mart-1	A*02:01	TCR	Melanoma
Tyrosinase	A*02:01	TCR	Melanoma
CEA	A*02:01	TCR	Solid cancers
Cancer germline			
NY-ESO-1	A*02:01	TCR	Solid cancers
PRAME	A*02:01	TCR	Solid cancers
MAGE-A4	A*02:01	TCR	Solid cancers
MAGEA4/8	A*02:01	TCR	Solid cancers
MAGE-A4	A*02:01	TCR-mimic	Solid cancers
MAGE-A10	A*02:01	TCR	Various
PRAME	A*02:01	TCR	Various
Overexpressed			
WT-1	A*02:01	TCR-mimic	AML
Viral antigens			
HPV16 E6	A*02:01	TCR	HPV16+ cancers
HPV16 E7	A*02:01	TCR	HPV16+ cancers
Neoantigens			
TP53 (R175H)	A*02:01	TCR	Breast
KRAS (G12D)	C*08:02/A*11:01	TCR	Pancreatic, NSC lung

Tissue Differentiation Antigens: Initial trials targeted non-mutated antigens like MART-1 and gp100 in melanoma, yielding antitumor responses but also on-target toxicities due to antigen expression in normal tissues.

Overexpressed Non-mutated Antigens: WT1 and p53, while not cancer-specific, have shown selective tumor targeting due to high expression differentials, though challenges like TCR-induced fratricide with p53 exist.

Cancer Germline Antigens (CGA): TCRs targeting NY-ESO-1, MAGE-A family, and PRAME have shown varied success, with some trials reporting high response rates but off-target toxicities in isoforms like MAGE-A3 due to homology with other normal proteins.

Viral Oncoproteins: Trials targeting viral oncoproteins, such as HPV E6/E7 and MCPyV, demonstrated tumor responses with limited off-target toxicity.

Private and Public Neoantigens: Personalized and public neoantigen-based TCRs, targeting mutations like KRASG12D and TP53, provide promise in durable responses, though resistance mechanisms such as HLA loss are noted.

Bispecific TCRs: Soluble bispecific TCRs (e.g., tebentafusp) offer therapeutic benefit in melanoma, suggesting potential for durable responses with manageable toxicity. However, their clinical progression may require extensive trials due to the survival-response uncoupling observed in early data.

The various trials with TCR or TCRL gene therapies have shown promising anti-cancer activity, though challenges with target selection, specificity, and toxicity highlight the need for precise antigen targeting and TCR engineering.

## 5 Focusing on the cellular protein landscape: utilizing TCR and TCR-like molecules in therapeutic strategies

### 5.1 TCRL-based soluble molecules

TCR-like mimic-based soluble molecules are a class of therapeutic agents that leverage the specificity of T-cell receptors (TCRs) to target and engage antigens with high precision. These molecules are designed to mimic the interaction between TCRs and their cognate antigens, but are not bound to a cell membrane like traditional TCRs. Instead, they exist in a soluble form, which offers unique advantages in terms of therapeutic application and flexibility. Here’s an overview of their key features and potential applications:

TCR-like mimics are engineered molecules that replicate the antigen-binding specificity of native TCRs. They are often designed to recognize specific peptide-MHC complexes. These mimics can be antibody molecules that are soluble and capable of binding to antigens in a manner similar to native TCRs. These soluble molecules function by binding to the target peptide-MHC complexes presented on the surface of cells. Their binding can block or modulate interactions that are typically recognized by TCRs, thereby influencing immune responses. In therapeutic contexts, they might be used to enhance or inhibit specific immune responses, depending on the desired outcome. TCR-like mimics are designed to recognize specific antigenic targets with high affinity and specificity, similar to how natural TCRs identify and bind to peptide-MHC complexes. Because they are soluble, TCR-like mimics can be engineered to bind a wide variety of targets, including those not typically accessible to membrane-bound receptors. This flexibility allows for targeting of a broader range of antigens. The solubility of these molecules may reduce the risk of off-target effects compared to membrane-bound TCRs, which can interact with a variety of cellular surfaces. Soluble molecules can often be produced more easily and at lower costs compared to membrane-bound TCRs, making them more accessible for therapeutic use. TCR-like mimics can be used to target tumor-specific antigens or neoantigens presented by cancer cells. By binding to these antigens, they can either stimulate a cytotoxic immune response or block signals that would otherwise suppress the immune response against the tumor. In autoimmune conditions, where the immune system erroneously attacks the body’s own tissues, TCR-like mimics could potentially be used to modulate or inhibit harmful immune responses.

Developing TCR-like mimics requires precise engineering to ensure that they bind with high specificity and affinity to their target antigens while avoiding off-target interactions. Ensuring the stability of soluble molecules in the body and effective delivery to the target site are critical for their therapeutic efficacy. While soluble molecules may have a lower risk of inducing immune responses compared to membrane-bound receptors, they still need to be carefully designed to minimize potential immunogenicity. Research is ongoing to improve the design, production, and application of TCR-like mimic-based soluble molecules. Advances in protein engineering, structural biology, and immunotherapy are expected to enhance their effectiveness and broaden their therapeutic applications. By harnessing the specificity of TCRs in a soluble format, these molecules offer a promising approach for targeted therapy across a range of diseases.

Targeting malignant cell intracellular proteome using extracellular recognition of pMHC complex by soluble molecules can be achieved using TCRL naked antibodies, soluble TCRs, armed TCRL-/TCR-based immunocytokines and bi-specific proteins; each tool provides a unique approach for tumor cell recognition and elimination (33, 43).

### 5.2 TCRL- and TCR-based soluble non-armed molecules

Soluble naked full IgG TCRL antibody can target and induce specific tumor cell elimination via several mechanisms, such as (a) antibody dependent cellular cytotoxicity (ADCC), where innate immune cells expressing Fc-gamma receptor binds the constant IgG region of the antibody, inducing specific lysis of the ab bounded target cell (33); (b) complement dependent cytolysis (CDC), where antibody bounded target cells undergo lysis via C5b-9 membrane attack complex (44); and (c) antibody-dependent phagocytosis (ADCP), mainly via target cell internalization and lysis by macrophages, mediated by Fc gamma receptor (45). Activation of both ADCC and CDC mechanisms was observed using a TCRL antibody, named 8F4, targeting PRI/HLA-A2 expressed on the cell surface of acute myeloid leukemia (AML) progenitor cells (34). Upon stability enhancement, soluble TCRs can also be used to target malignant cells. Several approaches were used to adjust the TCR native form to a stable soluble agent, such as random mutagenesis and computational modeling, aiming for surface hydrophobic residues’ replacement. Alternatively, fusion of the Ig constant domain to the TCRα and TCRβ extracellular domain, jun-fos leucine zipper fusion to the TCR C terminus, or novel addition of the interchain disulfide-bridge were found to improve soluble TCR stability (46). Several different soluble TCR molecules, mutated with an intradomain disulfide bond, showed superior stability in comparison with non-mutated soluble TCRs and maintained binding specificity toward pMHC-expressing target cells (47).

### 5.3 Bi-specific TCRL- and TCR-based soluble molecules

Malignant and immune cells dual targeting, can be achieved using bi-specific agents. In this approach, TCRL- or TCR-based molecules, targeted toward pMHC presented on malignant cells, can be fused to an anti-CD3 effector moiety, redirecting T cells against target malignant cells (34). Recently, a TCR-based bi-specific molecule, termed KIMMTRAK by Immunocore, targeted toward the malignant cell surface marker gp100/HLA-A2, fused to an anti-CD3 effector domain, was approved by the FDA for the treatment of unresectable or metastatic uveal melanoma, showing clinical benefit in a phase III clinical trial, via recruitment of T cells to gp100/HLA-A2 positive cells (48, 49). Of note, this drug is currently under phase 1/2 clinical trial for the treatment of Cutaneous melanoma (50). Several pMHC binding bi-specific molecules are currently under clinical trials, such as IMA401 and IMA202 by Immatics, targeting MAGE-A4/8 and MAGE-A1 in the context of HLA-A2, respectively (51), and HLA-A2-WT1 X CD3 bi-specific molecule for the treatment of adult acute myeloid leukemia by Roche/Genentech (52).

### 5.4 Armed TCRL- and TCR-based soluble molecules

Selective delivery of cytotoxic agents to tumor cells can be achieved using antibody–drug conjugates (ADCs), which can induce specific apoptosis of target cells, reducing the risk and severe side effects of general administration of cytotoxic materials (53). Such a TCRL armed molecule was tested against MART-1/HLA-A201 melanoma positive cells, delivering the PE38KDEL toxin to malignant target cells. This immunotoxin was found to significantly and specifically inhibit human melanoma growth in severe combined immunodeficient mice (54). Another immunotoxin, targeting the upregulated P53 peptide presented in the context of HLA-A24 on malignant cells, conjugated to a toxic DNA alkylating agent was found to limit tumor growth in NSG xenograft model (55).

### 5.5 TCR-engineered T cells

Engineered T cells, expressing a specific TCR, can be used for recognition and elimination of malignant cells, eliciting cytotoxic activity in response to pMHC-expressing target cells (56). The functional avidity of engineered T cells is a crucial measurement in the selection process of a TCR, assessed by the *in vitro* response of TCR-engineered T cell to different concentrations of the pMHC target epitope. *In vitro* assays determining functional avidity include the EC50 peptide concentration required for cytokine secretion, cytotoxic activity, and T cell proliferation (57). Functional avidity was previously shown to be improved using either TCR framework mutations or co-transfection of the TCR with the four CD3 chains (58). TCR affinity, measured by the interaction strength between pMHC and a single TCR, is also an important parameter in TCR candidate selection, as very high affinity TCRs may have impaired function owing to intense but short-lived response, while low TCR affinity may result in superior anti-tumor activity (59, 60). The relative expression of target epitope on malignant cells, termed antigen density, can also influence TCR activity, as relatively high antigen density results in impaired T cell activity (61). Additional challenge in TCR engineering is the concern for inappropriate α/β TCR chains pairing, as transduced T cells also express their native TCR, which may lower the desired TCR expression and, as a consequence, lower treatment efficiency. Several groups developed tools to address this problem, For example, Cohen et al. showed that desired TCR pairing can be achieved using engineered TCRs with constant murine regions, which preferably bind each other rather than the endogenous human TCR chains. Alternatively, the addition of a second disulfide bond to the transduced TCR can also improve the desired TCR pairing (62, 63). Another approach is to construct a single-chain TCR, which consists of both of the variable α and β TCR regions, connected by a linker (64). Extensive work in the TCR-engineered T cell field resulted in the first documented TCR-based clinical trial, targeted against melanoma antigen recognized by T cells 1 (MART-1), showing promising results, including tumor regression. These encouraging results led to the development of several anti-cancer TCR-based treatments, against targets such as gp100, NY-ESO-1, and AFP (65).

### 5.6 TCRL-based CAR engineered T cells

CAR-T cells are synthetic antigen-specific constructs expressed on lymphocytes, programmed to recognize and induce cancer cell death. Classical CAR construct consists of an extracellular domain, which is usually based on an ScFV structure. This region is composed of a short flexible linker, connecting the VH and VL domains of a monoclonal antibody, dictating CAR specificity toward a desired antigen. Traditional CAR constructs target MHC independent epitopes on the cell surface of malignant cells, such as the 2017 FDA approved anti-CD19 CAR-T cell therapy, showing remarkable success against B cell malignancies (66, 67). Expending CAR-T targets to intracellular epitopes can be achieved using TCRL-based CAR-T cells, directed against MHC dependent neoepitopes, based on the VH and VL regions of a TCRL antibody (68). As in TCR-engineered T cells, CAR-T activity depends on several factors, such as CAR-T avidity, affinity, and the antigen density, expressed by target malignant cells, all influencing CAR-T efficiency in tumor elimination. Comprehensive research, examining the influence of affinity, avidity, and antigen density using TCRL-based CAR-T cells, targeting Tyr/HLA-A2 antigen, was recently published by our group. In this study, we found that, as in TCR activity, very high antigen density results in diminished CAR-T activity, measured by cytokine production and CD107a upregulation. We also found that an optimal CAR-T response correlates with native TCR activity, at an antigen range of approximately 107–105 M, showing similar results when evaluating CAR-T with different avidities and affinities (69). Interestingly, cumulative results suggest that maximal CAR-T activity does not correlate with high affinity, as CAR-T cells with intermediate affinities (16–35 nM) showed an improved T cell response (69, 70). Currently, there are no public documented clinical trials examining the efficacy of TCRL-based CAR-T cells, but extensive work can be found in pre-clinical studies, such as the CAR-T targeting PR1/HLA-A2 epitope, derived from leukemia-associated antigen proteinase 3 and neutrophil elastase, showing specific activity against primary AML blasts, in an HLA-A2-dependent manner (71). Another example is the anti-melanoma CAR-T construct targeting NY-ESO-1 peptide in the context of HLA-A2. This CAR was tested for the treatment of melanoma implanted mouse model, resulting in delayed tumor progression. Of note, CAR targeting domain was originally based on an anti-NY-ESO-1/HLA-A2 Fab, which apparently, owing to high avidity, was found to lose specificity toward PR1/HLA-A2 epitope as a CAR, showing activity against non-peptide dependent HLA-A2 positive cells. Based on a crystal structure, specific mutation implemented to lower construct affinity toward the HLA-A2 epitope successfully resulted in a specific anti NY-ESO-1/hla-a2 CAR construct (72). Additional TCRL-based CAR-T examples targeting hematological malignancies and solid tumors can be found against the intracellular onco-protein WT1, specific liver cancer marker alpha-fetoprotein (AFP), and gp100, all showing promising results in pre-clinical studies (73–75).

## 6 The challenges of TCR-based therapeutic strategies

TCR or TCRL-based therapies (TCR-T) used as CAR-T or BiTE approaches are both innovative approaches in the realm of immunotherapy, but they differ significantly in their mechanisms and applications. TCR T therapy utilizes genetically engineered T cells that express a receptor capable of recognizing specific peptide fragments presented by Major Histocompatibility Complex (MHC) molecules on the surface of tumor cells. This allows TCR T cells to target intracellular antigens, making them potentially effective against a broader range of tumors, including those expressing mutated proteins or neoantigens (76–79). In contrast, CAR T therapy involves the engineering of T cells to express a synthetic receptor that directly targets surface antigens on tumor cells, independent of MHC presentation. This feature enables CAR T cells to efficiently recognize and eliminate tumors that may evade TCR recognition due to low MHC expression. Moreover, CAR T therapy has gained more clinical success in hematologic malignancies, such as certain leukemias and lymphomas, while TCR T therapy is still largely in the experimental stage, with ongoing research aimed at improving its efficacy and safety. Furthermore, the persistence and functionality of the modified T cells can vary significantly between the two therapies, with CAR T cells often leading to robust initial responses but facing challenges related to durability and resistance, while TCR T cells may have the potential for more sustained activity against a wider range of tumor types, though they can be limited by the availability of suitable target antigens and the risk of off-target effects. Overall, while both therapies share the goal of harnessing the immune system to fight cancer, their distinct targeting mechanisms and developmental trajectories underscore the complexity and potential of cancer immunotherapy (76–79).

A significant challenge in the application of TCR-T approaches lies in the specificity of TCRs and TCRL antibodies, particularly due to the complex landscape of tumor-associated antigens. Tumors can express neoantigens or aberrantly glycosylated proteins, which may lead to cross-reactivity with normal tissues, thereby causing off-target effects and limiting therapeutic efficacy. Specificity Challenges for TCR-based therapeutic strategies include: (i) Cross-Reactivity: One of the most significant challenges with TCRL antibodies is cross-reactivity. These antibodies may inadvertently bind to similar peptide-MHC complexes that are not the intended target. This is particularly concerning in cancers where antigens may share sequences with normal tissue, leading to potential autoimmune responses. (ii) Peptide Variability: The diversity of peptide presentation by MHC molecules complicates the specificity of TCRL antibodies. Variations in peptide sequences due to mutations or polymorphisms can affect binding affinity and specificity, making it difficult to predict which patients will benefit from a particular TCRL therapy. (iii) MHC Polymorphism: Human MHC molecules are highly polymorphic, leading to variations in peptide binding across different individuals. This variability necessitates the development of personalized TCRL antibodies, which can be resource-intensive and complex. (iv) Receptor Conformation: The binding conformation of TCRL antibodies can influence their specificity. Subtle changes in the structure of the antibody or the peptide-MHC complex can lead to differences in binding dynamics, potentially resulting in reduced specificity. (v) Tumor Microenvironment: The tumor microenvironment can modulate the expression of MHC molecules and peptides, further complicating the specificity of TCRL antibodies. Factors such as hypoxia, metabolic changes, and immune cell infiltration can alter antigen presentation, impacting the effectiveness of TCRL therapies.

To overcome these challenges, several strategies can be employed: Engineering of TCRL Antibodies: Advanced techniques such as phage display and rational design can help create TCRL antibodies with improved specificity. By selecting for antibodies that show minimal cross-reactivity in preclinical models, researchers can enhance the likelihood of successful therapeutic outcomes; Personalized Medicine Approaches: Tailoring TCRL antibodies to individual patients based on their specific MHC profiles and tumor antigen expression can improve specificity and reduce the risk of off-target effects; Combination Therapies: Using TCRL antibodies in conjunction with other therapies (e.g., checkpoint inhibitors) may help to enhance specificity while also improving overall therapeutic efficacy.

While TCRL antibodies represent a significant advancement in targeted cancer therapies, addressing the specificity challenges they present is critical for their successful clinical application. Continued research and innovative strategies will be essential to unlock the full potential of TCRL antibodies, ensuring that they can effectively target tumors while minimizing adverse effects on normal tissues. As the field progresses, refining these approaches will be key to improving patient outcomes in cancer immunotherapy.

Another challenge in the field of TCR-T lies with the observation that tumors can employ various immune escape mechanisms that diminish the efficacy of these therapies (80–85). The understanding of immune escape mechanisms and strategies to overcome these escape mechanisms and enhance the effectiveness of TCR-based therapies is essential for future advance in this therapeutic area.

Tumor cells can evade TCR-mediated recognition through several mechanisms (80–85), including:

Antigen Loss or Alteration: Tumors may downregulate or mutate antigens recognized by TCRs, leading to reduced visibility to T cells; Immunosuppressive Tumor Microenvironment: The presence of regulatory T cells (Tregs), myeloid-derived suppressor cells (MDSCs), and immunosuppressive cytokines can inhibit T cell activation and function; Checkpoint Molecule Upregulation: Tumor cells may express immune checkpoint molecules like PD-L1, which can inhibit T cell activity; and HLA Loss: Downregulation of major histocompatibility complex (MHC) molecules can prevent T cell recognition of tumor cells.

Strategies to Enhance TCR Therapy Efficacy may include: Targeting Multiple Antigens: Developing TCRs that target multiple antigens can reduce the likelihood of tumor cells escaping recognition through antigen loss. This multi-target approach may involve the use of combination therapies with checkpoint inhibitors to enhance overall efficacy. Enhancing T Cell Persistence and Function: Engineering T cells to express cytokines or survival factors can improve their persistence in the tumor microenvironment. For example, incorporating genes for IL-15 or other supportive cytokines can enhance T cell expansion and longevity. Combining TCR Therapies with Immune Checkpoint Inhibitors: The concurrent use of TCR-based therapies with checkpoint inhibitors (e.g., anti-PD-1/PD-L1) can help overcome immunosuppressive signals and reinvigorate exhausted T cells, boosting anti-tumor responses. Modulating the Tumor Microenvironment: Strategies to alter the tumor microenvironment, such as targeting Tregs or MDSCs, can create a more favorable setting for T cell activity. Agents that deplete or inhibit these immunosuppressive cells can enhance TCR therapy effectiveness. Utilizing Oncolytic Viruses: Oncolytic viruses can selectively infect and kill tumor cells while also inducing an immune response against tumor antigens. Combining these therapies with TCRs may enhance antigen presentation and overcome immune escape. Gene Editing Technologies: CRISPR/Cas9 and other gene-editing tools can be employed to modify T cells to improve their functionality, such as knocking out genes responsible for checkpoint regulation or enhancing TCR specificity. Personalized TCR Engineering: Utilizing neoantigens specific to an individual’s tumor for TCR design can increase the likelihood of effective targeting. This personalized approach can circumvent antigen loss by focusing on unique tumor markers.

Overcoming immune escape mechanisms is critical for enhancing the efficacy of TCR-based therapies. The integration of innovative strategies, including multi-antigen targeting, combination therapies, and modulation of the tumor microenvironment, holds great promise. Continued research and clinical trials will be essential to validate these approaches and optimize TCR therapies for improved patient outcomes in cancer treatment. As our understanding of tumor immunology evolves, these strategies will be pivotal in the fight against cancer.

## 7 Concluding remarks

The exploration of the intracellular proteome for immunotherapy represents a transformative frontier in cancer treatment, bridging the gap between molecular biology and clinical application. Throughout this review, we have highlighted the pivotal role of intracellular proteins in shaping immune responses and their potential as therapeutic targets.

The recent advancements in proteomics and our understanding of the intracellular proteome have unveiled a wealth of potential targets for immunotherapy. Techniques such as mass spectrometry and advanced bioinformatics have provided unprecedented insights into the protein composition of cancer cells and their interactions with the immune system. These discoveries have laid the groundwork for novel therapeutic strategies that can potentially overcome the limitations of conventional treatments.

Despite the promising developments, targeting the intracellular proteome poses significant challenges. The inherent complexity of the intracellular environment, the variability in protein expression, and the potential for off-target effects require careful consideration. Additionally, the dynamic nature of protein-protein interactions and post-translational modifications adds layers of complexity that must be addressed to design effective and specific immunotherapies.

The integration of intracellular proteome targeting into immunotherapy holds the potential to revolutionize cancer treatment. By exploiting the unique protein signatures of cancer cells and harnessing the immune system’s ability to recognize and respond to these targets, we can advance towards more effective and less toxic treatment modalities.

Although pMHC targeting moieties have shown promising results in tumor elimination, there remains limited information on their potential in preclinical and clinical studies. Therefore, TCRL- and TCR-based soluble molecules, engineered TCRs, and TCRL-based CAR-T cells should be further evaluated for their safety and potential for unpredictable cross-reactivity toward non-malignant pMHC-expressing cells (86). Another challenge in targeting pMHC epitopes is the identification of common neo-peptides that are shared among a broad patient population. This limitation may be mitigated through experimental analysis and further characterization of prevalent pMHC complexes (87). Additionally, immune escape mechanisms, such as the downregulation of pMHC expression in malignant cells, must be considered when treating patients with pMHC-targeting molecules (88). Despite these challenges, current literature provides compelling evidence and future promise for TCRL- and TCR-based therapies as potential new modalities in cancer immunotherapy.

T cell receptor (TCR T) therapy and chimeric antigen receptor T cell (CAR T) therapy are both novel groundbreaking approaches within immunotherapy but differ significantly in their mechanisms, applications, and current clinical impact. TCR T therapy involves genetically engineered T cells that express receptors specifically designed to recognize peptide fragments presented by Major Histocompatibility Complex (MHC) molecules on tumor cells. This mechanism allows TCR T cells to target intracellular antigens, making them suitable for recognizing a broad array of tumor-specific targets, including mutated proteins and neoantigens. These characteristic holds promise for addressing tumor types that present intracellular antigens, broadening potential applications to cancers that exhibit such molecular signatures. In contrast, CAR T therapy involves engineering T cells to express synthetic receptors that directly target specific surface antigens on tumor cells, bypassing the need for MHC-mediated antigen presentation. This attribute allows CAR T cells to efficiently detect and attack tumors that might evade TCR-based recognition due to low or variable MHC expression—a common mechanism tumors use to evade immune detection. Clinically, CAR T therapy has demonstrated remarkable success, particularly in hematologic malignancies like certain leukemias and lymphomas, where CAR T cell products are now approved and in use. However, TCR T therapy remains largely in experimental stages, with active research underway to enhance its efficacy and safety, especially in solid tumors (89–91).

The persistence, functionality, and durability of these engineered T cells also differ. CAR T cells tend to show robust initial responses in many cases but face challenges related to long-term persistence, exhaustion, and resistance, which can limit durability in the fight against certain cancers. TCR T cells, on the other hand, may offer more sustained activity, potentially extending the therapy’s benefits over a longer period. However, TCR T’s success depends heavily on the availability of suitable target antigens that are both tumor-specific and safely targeted without causing off-tumor effects, a challenge that underscores the risk of off-target effects in TCR T therapies.

Overall, while TCR T and CAR T therapies share the objective of harnessing the immune system to combat cancer, their distinct targeting mechanisms, technical challenges, and developmental trajectories underscore the complexity and promise of advanced immunotherapies. As research continues, these differences may offer complementary strategies in the future, with each approach being optimized for different tumor types and clinical contexts.

Future research should focus on refining techniques to precisely target and modulate specific proteins within the intracellular milieu. Integrating systems biology approaches with experimental validation can enhance our understanding of the proteome and its role in immune evasion and tumor progression. Moreover, personalized medicine approaches, leveraging patient-specific proteomic profiles, could lead to more tailored and effective therapies.

In summary, the journey towards targeting the intracellular proteome is both challenging and promising. Continued research and innovation in this field will be crucial for translating these insights into clinical practice, ultimately improving outcomes for patients with cancer and other diseases where traditional therapies have fallen short.
